# Supplementary material for: Impaired therapeutic efficacy of bone marrow cells from post-myocardial infarction patients in the TIME and LateTIME clinical trials
Source: PLoS One. 2020 Aug 25;15(8):e0237401. doi: 10.1371/journal.pone.0237401 (PMC7446972; doi:10.1371/journal.pone.0237401)
Supplement: S1 Table — (DOCX) [file pone.0237401.s001.docx]

**Supporting information**

**S1 Table**. **Subject information for implantation of bone marrow mononuclear cells**

Group 1 Group 2

Condition Healthy TIME LateTIME Healthy TIME

(n=6) (n=6) (n=6) (n=3) (n=6)

Gender

Male 5 6 6 2 6

Female 1 0 0 1 0

Age (year) 43.3 ± 6.4 46.0 ± 4.67 47.41 ± 2.16 44.3 ± 5.1 46.0 ± 4.67
