# Supplementary material for: Impaired therapeutic efficacy of bone marrow cells from post-myocardial infarction patients in the TIME and LateTIME clinical trials
Source: PLoS One. 2020 Aug 25;15(8):e0237401. doi: 10.1371/journal.pone.0237401 (PMC7446972; doi:10.1371/journal.pone.0237401)
Supplement: S2 Table — Data are means ± SD. EDV, end-diastolic volume; EF, ejection fraction; ESV, end-systolic volume; MI, myocardial infarction; MNCs, mononuclear cells; HBSS, Hanks balanced salt solution. Statistically significant differences between all groups were shown in Figs 1 and 2. (DOCX) [file pone.0237401.s002.docx]

**Supporting information**

**S2 Table**. **Recipient echocardiographic parameters pre- and post-MI**

Donor condition EF (%) ESV (μl) EDV (μl)

*Figure 1*

Healthy-MNCs (n=7)

Baseline 51.6 ± 0.9 20.5 ± 2.4 42.3 ± 4.7

2d post-MI 33.9 ± 1.8 25.3 ± 2.8 38.3 ± 4.5

28d post-MI 40.1 ± 7.7 31.4 ± 8.5 51.8 ± 8.2

LateTIME-MNCs (n=10)

Baseline 51.6 ± 1.2 20.5 ± 1.6 42.3 ± 2.6

2d post-MI 33.6 ± 2.3 27.4 ± 6.3 41.2 ± 9.4

28d post-MI 30.1 ± 7.7 44.1 ± 11.9 62.2 ± 10.8

TIME-MNCs (n=7)

Baseline 51.2 ± 0.8 21.1 ± 1.1 43.3 ± 2.2

2d post-MI 33.3 ± 2.1 25.6 ± 4.2 38.4 ± 5.5

28d post-MI 28.1 ± 9.7 48.3 ± 25.0 64.8 ± 24.3

HBSS (n=6)

Baseline 51.7 ± 1.4 20.6 ± 1.2 42.6 ± 1.4

2d post-MI 32.4 ± 2.0 25.7 ± 7.2 37.9 ± 10.1

28d post-MI 26.5 ± 11.2 48.9 ± 14.5 65.7 ± 11.0

*Figure 2*

Healthy MNCs (n=8)

Baseline 50.1 ± 1.2 36.4 ± 1.3 72.9 ± 2.9

2d post-MI 31.5 ± 4.5 39.6 ± 5.4 57.7 ± 5.8

21d post-MI 36.4 ± 4.6 45.8 ± 7.5 71.8 ± 8.4

28d post-MI 36.2 ± 4.0 46.7 ± 7.5 73.2 ± 9.6

MI MNCs (n=10)

Baseline 50.0 ± 3.4 34.8 ± 5.4 69.3 ± 7.1

2d post-MI 31.4 ± 4.1 41.0 ± 7.3 59.4 ± 8.0

21d post-MI 25.1 ± 4.3 61.5 ± 16.1 81.6 ± 17.9

28d post-MI 23.1 ± 4.9 69.8 ± 20.5 90.0 ± 22.6

Data are means ± SD. EDV, end-diastolic volume; EF, ejection fraction; ESV, end-systolic volume; MI, myocardial infarction; MNCs, mononuclear cells; HBSS, Hanks balanced salt solution. Statistically significant differences between all groups were shown in Figs 1 and 2.
